# Prevalence and associated factors of depression, anxiety, and stress among university athletes in Bangladesh

**DOI:** 10.1007/s44192-026-00660-9

**Published:** 2026-11-06

**Authors:** Kazi Md Azman Hossain, Jannatul Ferdous Rikti, Mehedi Hasan Badsha, Abdul Al Raihan, Touhidul Islam Badhon, Md. Emon Hossen Mondol, Md. Jamil Hossain Joy, Sree Pallob, Al Amin

**Affiliations:** 1 Department of Physiotherapy and Rehabilitation, Jashore University of Science and Technology (JUST), Jashore, 7408 Bangladesh; 2 Dhaka College of Physiotherapy, Dhaka, Bangladesh

**Keywords:** Anxiety, Depression, Lifestyle factors, Stress, University athletes

## Abstract

**Background:**

University athletes face substantial academic, physical, and psychosocial pressures that may heighten vulnerability to psychological distress, despite the perceived mental health benefits of sports participation. This study aimed to determine the prevalence of depression, anxiety, and stress among university athletes in Bangladesh and to examine how these conditions are associated with lifestyle and sociodemographic factors.

**Methods:**

This cross-sectional study was conducted from March to September 2025 among 249 university athletes aged 18–26 years at six randomly selected universities in Bangladesh. Data were collected online and in person. Psychological distress was assessed with the Depression, Anxiety, and Stress Scale-21 (DASS-21), and health-promoting lifestyle behaviors were evaluated with the Health-Promoting Lifestyle Profile II (HPLP-II). Descriptive statistics, chi-square tests, Mann–Whitney U tests, Spearman’s correlations, and binary logistic regression analyses were conducted.

**Results:**

The prevalence of depression, anxiety, and stress was 42.6%, 42.6%, and 43.8%, respectively. Depression, anxiety, and stress were significantly associated with relationship status, fatigue, socioeconomic status, smoking, sleep duration, sports participation frequency, and satisfaction with sports (all *p* < 0.05). Participants with depression, anxiety, and stress had significantly lower scores across all HPLP-II domains (all *p* < 0.01). Higher physical activity was independently associated with lower odds of depression (OR = 0.462, 95% CI: 0.286–0.746), anxiety (OR = 0.756, 95% CI: 0.677–0.845), and stress (OR = 0.773, 95% CI: 0.677–0.882). Better stress management and stronger interpersonal relationships were also significantly associated, whereas other factors were not.

**Conclusions:**

Depression, anxiety, and stress were highly prevalent among university athletes in Bangladesh and strongly associated with modifiable lifestyle factors. These findings support integrated, athlete-centered mental health strategies emphasizing physical activity, stress management, and psychosocial support. However, the cross-sectional design precludes causal inference and determination of the directionality of these associations.

**Clinical trial number:**

Not applicable.

**Supplementary Information:**

The online version contains supplementary material available at https://doi.org/10.1007/s44192-026-00660-9.

## Introduction

Mental health is a fundamental component of overall well-being and a key determinant of individual functioning and societal development [1]. Despite growing global recognition of psychological distress (PD), including symptoms of depression, anxiety, and stress, it remains a substantial public health concern and contributes significantly to the global burden of disease [2]. Epidemiological evidence indicates that mental disorders affected approximately 12% of the global population in 2019 and accounted for nearly 5% of total disability-adjusted life years (DALYs), underscoring their widespread impact [2, 3]. Depression, anxiety, and stress frequently co-occur and can adversely affect cognitive functioning, academic performance, social relationships, and quality of life. University students may be particularly vulnerable to PD because of academic demands, social adjustment, financial pressures, evolving interpersonal relationships, and the transition toward greater independence [3–5].

University athletes represent a distinct subgroup within this population because they must navigate academic responsibilities and sport-specific demands simultaneously. Intensive training, competition, performance expectations, injury risk, and time constraints may impose additional psychological demands [6, 7]. Recent international evidence indicates that training load, academic pressure, and coach-athlete or team dynamics are important stressors among university athletes, with anxiety, depressive symptoms, burnout, and other forms of PD reported across diverse sporting contexts [8, 9]. These findings indicate that university athletes’ mental health should be considered within their combined academic and sporting environment rather than inferred solely from evidence involving general university students. However, relatively little research has examined PD among university athletes in relation to multidimensional health-promoting lifestyle behaviors, particularly in low- and middle-income settings such as Bangladesh.

Health-promoting lifestyle behaviors may be an important, potentially modifiable component of this broader environment. Physical activity, nutrition, stress management, interpersonal relationships, and other health-promoting behaviors may influence psychological well-being through interconnected behavioral, physiological, and psychosocial pathways [5, 6, 8]. Regular physical activity may support mood regulation, stress adaptation, sleep quality, and self-efficacy, whereas healthy dietary practices may contribute to physiological and metabolic well-being relevant to psychological functioning. Effective stress management may strengthen coping and emotional regulation, while positive interpersonal relationships may enhance perceived social support and resilience. These pathways are unlikely to operate independently; rather, health-promoting behaviors may cluster into broader behavioral patterns with potentially complementary effects on mental health. Consistent with this perspective, recent systematic evidence indicates that healthier combinations of lifestyle behaviors are associated with fewer symptoms of depression, anxiety, and stress. Evidence among university populations also suggests beneficial associations between physical activity and mental health outcomes [10]. However, these relationships may be bidirectional, as PD can itself influence motivation, sleep, physical activity, dietary practices, and social engagement. Accordingly, associations observed in cross-sectional research should not be interpreted as evidence of causality.

This multidimensional perspective may be particularly relevant to university athletes, whose lifestyle behaviors are shaped by the competing demands of academic study and athletic participation. Recent literature has increasingly emphasized athlete-centered approaches to mental health promotion and prevention, highlighting interactions among training demands, academic obligations, social relationships, and psychological well-being [8, 9]. Nevertheless, much of the existing research has examined individual risk or protective factors, specific athlete subgroups, or general university populations. Such approaches may provide an incomplete representation of the behavioral context in which PD occurs. Assessing multiple health-promoting lifestyle domains within a unified framework may therefore provide a more comprehensive and ecologically relevant understanding of factors associated with PD among university athletes.

In Bangladesh, existing evidence indicates a substantial burden of depression, anxiety, and stress among university students, with previous studies identifying associations with sociodemographic, academic, health, and behavioral characteristics [11–13]. However, this literature has predominantly focused on general university student populations, providing limited evidence specific to university athletes, who face additional sport-related demands. Furthermore, previous Bangladeshi studies have generally examined individual behavioral or sociodemographic characteristics rather than comprehensively assessing multiple health-promoting lifestyle domains. The Health-Promoting Lifestyle Profile II (HPLP-II) provides a validated multidimensional framework encompassing health responsibility, physical activity, nutrition, interpersonal relations, spiritual growth, and stress management, allowing these domains to be evaluated within a unified framework. Thus, an important evidence gap remains regarding the burden of PD among university athletes in Bangladesh and its relationship with multidimensional health-promoting lifestyle behaviors.

The present study therefore addresses a population-, contextual-, and conceptually specific gap by focusing on university athletes in Bangladesh rather than extrapolating findings from general university student populations. It aimed to determine the prevalence of depression, anxiety, and stress among university athletes in Bangladesh and to examine their associations with multidimensional health-promoting lifestyle factors measured using the HPLP-II, while considering relevant sociodemographic characteristics. By examining multiple lifestyle domains simultaneously within an athlete-specific population and Bangladesh-specific context, this study seeks to provide contextually relevant evidence on potentially modifiable lifestyle factors associated with PD and to inform athlete-centered mental health promotion within university settings.

## Methods

### Study design, sampling approach, and sample size estimation

This cross-sectional study examined the prevalence of depression, anxiety, and stress among university athletes in Bangladesh aged 18–26 years and explored lifestyle and sociodemographic factors associated with these outcomes. A two-stage sampling strategy was used, with simple random sampling of universities at the first stage and convenience sampling of eligible university athletes at the second stage. Thus, although universities were randomly selected, individual participants were not selected using probability sampling.

**Fig. 1:**
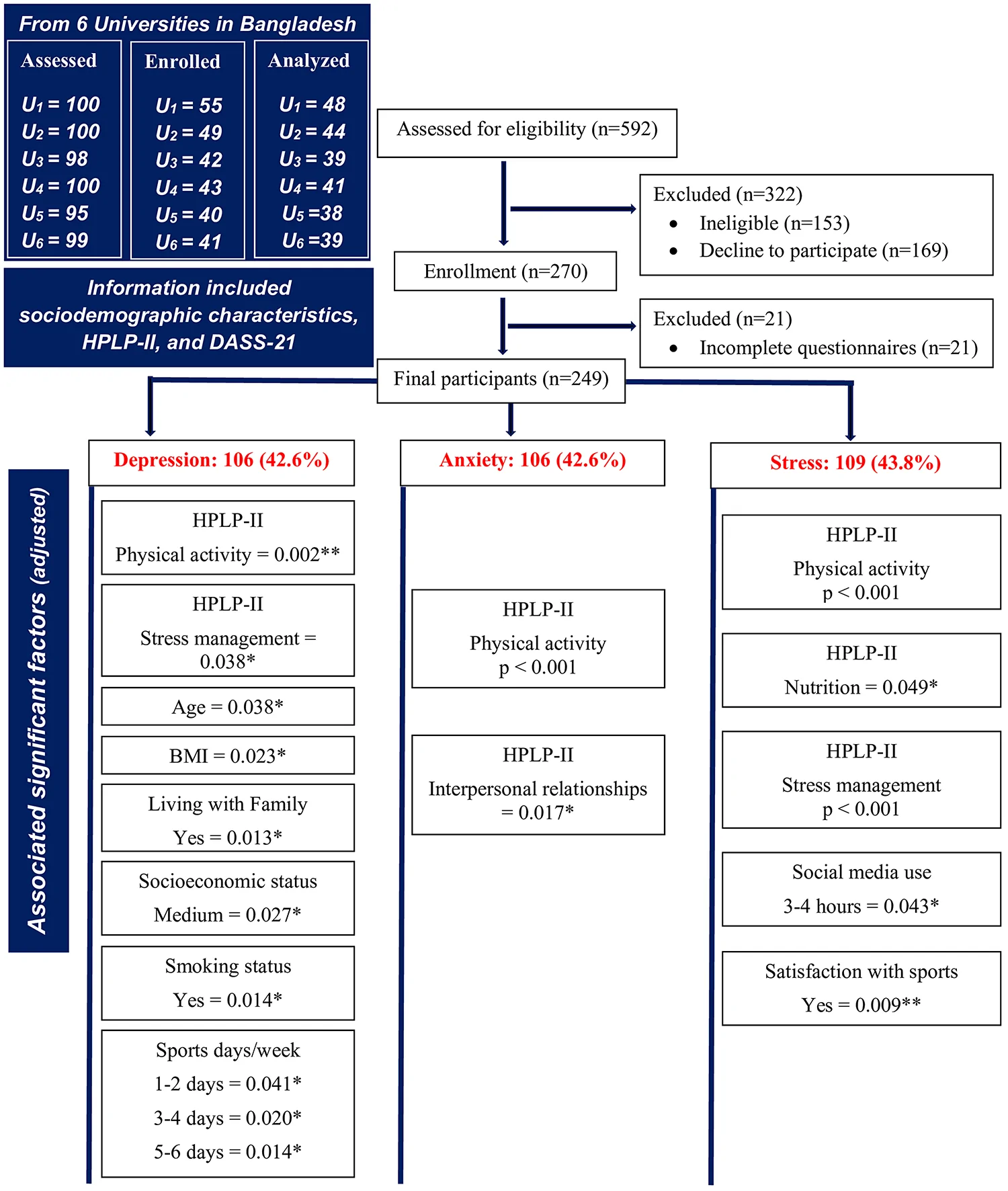
Study overview. *p<, **p<0.01, ***p<0.001. HPLP-II: Health Promoting lifestyle Profile-II; BMI: Body Mass Index; DASS: Depression, Anxiety and Stress Scale

The required sample size was estimated using the single-proportion formula [14]:

$${\rm{n = }}{{\rm{Z}}^2}{\rm{p}}\left( {{\rm{1 - p}}} \right){\rm{/}}{{\rm{d}}^2}$$

where n represents the required sample size, Z is the standard normal deviate corresponding to a 95% confidence level (1.96), p is the anticipated prevalence, and d is the desired absolute precision of the prevalence estimate. We used an anticipated prevalence of 20% as an a priori planning assumption based on the literature available during study planning [9, 10]. Because directly comparable prevalence estimates among Bangladeshi university athletes were limited, this value was used as a pragmatic planning estimate rather than as a precise estimate of the expected prevalence in the study population. Using *p* = 0.20 and an absolute precision of 5%, the calculated minimum sample size was 246 participants. We set an intended recruitment target of approximately 270 participants to allow for potential non-response and incomplete questionnaires. During data collection, 270 questionnaires were obtained; 21 were excluded because of substantial missing or incomplete responses, resulting in 249 participants in the final analysis. The sample-size calculation was specified before data analysis and was not modified based on the observed prevalence. Figure 1 represents the overview of this study.

### University selection and participant recruitment

For the first sampling stage, a sampling frame was established before recruitment by compiling a complete list of universities that met the study eligibility criteria and were accessible to the research team during the planned data-collection period. The list was entered into Microsoft Excel, and the RAND() function was used to generate random numbers for the institutions. Six universities were selected by simple random sampling, giving each university in the sampling frame an equal probability of selection.

For the second stage, eligible university athletes within the selected universities were recruited using convenience sampling. Recruitment was undertaken among students who met the prespecified eligibility criteria and were available and willing to participate between March and September 2025. Individual athletes were therefore not randomly sampled, and a complete sampling frame of eligible university athletes was not available. Participants were approached through institutional and student communication channels for online recruitment and through direct contact during visits to the selected universities for face-to-face recruitment. Participation was voluntary, and students were not required to participate as part of their academic or sporting activities.

The use of convenience sampling reflected practical constraints related to obtaining complete lists of eligible university athletes and accessing individual athletes across the selected universities within the available time and resources. Efforts were made to recruit participants with diverse sociodemographic and sporting characteristics; however, these measures do not eliminate the possibility of selection bias. Accordingly, the final sample should not be interpreted as a probability-based representative sample of university athletes in Bangladesh, and the generalizability of the findings beyond the participating universities should be considered with appropriate caution.

### Data collection procedure

Data were collected between March and September 2025 using both online self-administration and face-to-face administration to broaden access to eligible university athletes across the selected universities. We applied the same eligibility criteria, questionnaire, and study procedures across both recruitment modes.

For the online component, we developed a structured, self-administered questionnaire using Google Forms and disseminated it through institutional and student communication channels, including relevant WhatsApp groups. The questionnaire comprised three sections: (1) an introductory section describing the study purpose, eligibility criteria, voluntary participation, confidentiality, and informed-consent procedures; (2) a sociodemographic and health/lifestyle section incorporating the Health-Promoting Lifestyle Profile II (HPLP-II); and (3) the Depression Anxiety Stress Scale-21 (DASS-21) for assessing symptoms of depression, anxiety, and stress.

For the face-to-face component, trained data collectors visited the selected universities and approached eligible university athletes. Before data collection, data collectors received standardized training, including approximately 2 h of practice covering participant eligibility, questionnaire administration, informed consent, and data-recording procedures. The same standardized questionnaire was administered under supervision to maintain consistency across data collectors and recruitment sites.

Because the two recruitment modes represented different pathways for participant access, we recognized the possibility of differences in participant characteristics and mode-related selection bias. We did not conduct a prespecified comparison or sensitivity analysis between online and face-to-face participants. Therefore, we acknowledge the mixed-mode recruitment strategy as a potential source of selection and mode-related bias, and the findings should be interpreted accordingly.

To minimize duplicate participation, the online questionnaire was configured to allow only one submission per participant where technically feasible. For face-to-face recruitment, participant flow, recruitment locations, and collection times were coordinated to minimize the possibility of repeat participation. Participants were informed of the study objectives, confidentiality procedures, and voluntary nature of participation and provided informed consent before completing the questionnaire. Responses were collected anonymously; consequently, individual submissions could not be linked to participants after submission for subsequent withdrawal. This condition was communicated during the consent process.

### Measurement tools

The questionnaire comprised three sequential sections: (1) an introductory section describing the study purpose, eligibility criteria, confidentiality, and informed-consent procedures; (2) a sociodemographic and health/lifestyle section incorporating the Health-Promoting Lifestyle Profile II (HPLP-II); and (3) a psychological assessment section using the Depression Anxiety Stress Scale-21 (DASS-21) to assess symptoms of depression, anxiety, and stress. The questionnaire was initially developed in English and translated into Bengali using a forward–backward translation procedure conducted by two independent bilingual researchers to promote linguistic and conceptual equivalence.

The sociodemographic section was designed to capture a comprehensive participant profile. It included variables such as age (reported as mean ± SD and categorized into ≤ 20, 21–24, and ≥ 25 years), gender (male/female), Body Mass Index (BMI) - (kg/m²; mean ± SD and categorized as underweight, normal weight, overweight, and obese), relationship status (single, in a relationship, married), and living with family (yes/no). Additional indicators included perceived fatigue level (never, low, moderate, high), family socioeconomic status (low, medium, high), sports days/week (1–2/3–4/5–6/7 days), satisfaction with sports (yes/no), and key lifestyle behaviors such as smoking status (yes/no), daily social media use (≤ 2 h, 3–4 h, ≥ 5 h), and sleep duration (≤ 6 h, 7–8 h, ≥ 9 h).

Participants’ health-related lifestyle behaviors were assessed using the HPLP-II. The HPLP-II is a widely validated instrument designed to evaluate multidimensional health-promoting behaviors across six domains: health responsibility (9 items), physical activity (8 items), nutrition (9 items), spiritual growth (9 items), interpersonal relations (9 items), and stress management (9 items), comprising a total of 52 items. Each item is rated on a 4-point Likert scale from 1 (“never”) to 4 (“routinely”), indicating how frequently individuals engage in specific health-promoting behaviors. All items are positively worded, with total possible scores ranging from 52 to 208. Higher scores indicate a greater engagement in health-promoting lifestyle practices and, consequently, a more favorable overall health behavior profile. ICC = 0.89–0.92 [15, 16].

Psychological outcomes were assessed using the validated version of the Depression, Anxiety, and Stress Scale (DASS-21), a widely used 21-item self-report instrument comprising three subscales for depression, anxiety, and stress. Each item is rated on a four-point Likert scale reflecting symptoms experienced over the preceding week. Scores were interpreted using established cut-off values reported in previous research, with participants classified as “normal” or as exhibiting depression, anxiety, or stress indicators. For the prevalence and associated-factor analyses, DASS-21 scores were classified into binary categories to distinguish participants within the normal range from those reporting symptoms above the conventional normal-range threshold. Following the established DASS-21 scoring procedure, subscale scores were interpreted using the conventional cut-points: depression, 0–9 (normal) and > 9 (depressive symptoms); anxiety, 0–7 (normal) and > 7 (anxiety symptoms); and stress, 0–14 (normal) and > 14 (stress symptoms). These categorizations were used for subsequent statistical analyses. This binary classification aligned with the study objective of estimating the prevalence of symptom indicators and examining factors associated with their presence [17, 18]. ICCs = 0.78–0.92 [19].

The DASS-21 subscales demonstrated acceptable-to-excellent internal consistency in the present sample, with Cronbach’s α coefficients ranging from 0.723 to 0.906 across the Depression, Anxiety, and Stress subscales. Similarly, the six HPLP-II domains demonstrated acceptable-to-excellent internal consistency, with Cronbach’s α coefficients ranging from 0.779 to 0.825.

### Data cleaning and analysis

Following data collection, the dataset was systematically reviewed and cleaned using Microsoft Excel 2019 to assess completeness, consistency, and eligibility. The researchers applied predefined inclusion and exclusion criteria before analysis. Participants were eligible for inclusion if they had provided informed consent, were aged 18–26 years, were identified as university athletes from one of the selected universities, and had sufficiently complete questionnaire data for the planned analyses.

Responses with substantial missing, incomplete, or internally inconsistent information were excluded during data screening. The data-cleaning process included checking participant eligibility, completeness of questionnaire responses, consistency of recorded variables, and potential duplicate submissions. After verification, eligible records were retained for analysis, and the cleaned dataset was coded numerically and prepared for statistical analysis.

### Statistical analysis

All statistical analyses were conducted in SPSS version 24.0. Descriptive statistics, including means, standard deviations, and frequency distributions, summarized participants’ sociodemographic characteristics. Normality was assessed with the Kolmogorov–Smirnov test. Because the distributions did not meet the normality assumption (*p* < 0.05), nonparametric methods were used during the analysis. Associations between categorical variables and DASS-21 categories were examined with the chi-square test. Relationships between HPLP-II and DASS-21 scores were assessed with Spearman’s rank correlation. Group differences in HPLP-II scores across DASS-21 categories were analyzed with the Mann–Whitney U test. Binary logistic regression models were fitted separately for depression, anxiety, and stress to identify factors associated with each binary outcome. Candidate predictors were selected a priori based on their conceptual relevance to the outcomes and availability in the dataset. Categorical predictors were entered using indicator variables with prespecified reference categories. Associations were expressed as odds ratios (ORs) with 95% confidence intervals (CIs). Model adequacy was assessed using complementary diagnostics, including multicollinearity (variance inflation factor [VIF] and tolerance), the Hosmer–Lemeshow goodness-of-fit test, receiver operating characteristic (ROC) analysis with area under the curve (AUC), and the number of outcome events relative to the number of estimated model parameters. The distribution of observations across outcome categories was also examined for evidence of marginally sparse cells. The stability of coefficient estimation was further assessed by reviewing model convergence and the presence of unusually large standard errors or confidence intervals. Given the relatively high prevalence of the outcomes, ORs were interpreted as measures of association rather than estimates of risk or causal effects. Supplementary Table 1 reports the model diagnostics and discrimination measures. All tests were two-sided, with *p* < 0.05 considered statistically significant [16].

## Results

### Sociodemographic characteristics

The study included 249 university athletes. Participants represented soccer (*n* = 68) and cricket (*n* = 49), the highest numbers, followed by basketball (*n* = 35), sprinters (*n* = 30), tennis (*n* = 25), field hockey (*n* = 23), and swimming (*n* = 19), respectively, with a mean age of 21.97 ± 2.40 years. Most participants were aged 21–24 years (55.8%), male (58.6%), single (65.5%), and living with their families (69.1%). Most participants had a normal body mass index (74.3%) and belonged to a middle socioeconomic background (61.8%). Nearly half of the participants reported moderate fatigue (49.4%), while 47.8% were smokers. Regarding lifestyle factors, 47.0% used social media for 3–4 h daily, and 55.4% reported sleeping 7–8 h per day. A large proportion engaged in sports activities 5–6 days per week (41.0%) and were satisfied with their sports participation (71.9%). The prevalence of psychological distress indicators showed that depression and anxiety were present in 42.6% of participants, while stress was slightly higher at 43.8%, indicating a substantial burden of mental health concerns among university students.

### Depression

Depression was significantly associated in the bivariate analyses with relationship status, fatigue level, family socioeconomic status, smoking status, social media use, sleep duration, sports participation frequency, and satisfaction with sports (all *p* < 0.05). Participants classified with depressive symptoms had lower HPLP-II scores across all six lifestyle domains and lower total HPLP-II scores than those without depressive symptoms (95.03 ± 14.75 vs. 131.77 ± 18.22, *p* < 0.001). In the adjusted logistic regression model, older age was associated with higher odds of depressive symptoms (OR = 1.829, 95% CI: 1.035–3.232, *p* = 0.038), whereas higher BMI was associated with lower odds (OR = 0.534, 95% CI: 0.311–0.916, *p* = 0.023). Living with family (OR = 0.001, 95% CI: 0.000–0.208, *p* = 0.013) and medium socioeconomic status (OR = 0.003, 95% CI: 0.000–0.512, *p* = 0.027) were also associated with lower odds. Smoking was associated with substantially higher estimated odds of depressive symptoms (OR = 134.689, 95% CI: 2.740–6621.075, *p* = 0.014); however, the very wide confidence interval indicates considerable imprecision in this estimate. Compared with daily sports participation, participation on fewer days per week was associated with lower estimated odds of depressive symptoms (1–2 days: OR = 0.003, 95% CI: 0.000–0.777; 3–4 days: OR = 0.001, 95% CI: 0.000–0.357; 5–6 days: OR = 0.003, 95% CI: 0.000–0.316). Higher physical activity and stress-management scores were also associated with lower odds of depressive symptoms (OR = 0.462, 95% CI: 0.286–0.746; and OR = 0.723, 95% CI: 0.532–0.982, respectively). Given the extreme ORs and wide confidence intervals for several variables, interpret these estimates cautiously as associations rather than precise measures of effect.

### Anxiety

Anxiety was significantly associated in the bivariate analyses with relationship status, fatigue, family socioeconomic status, smoking status, sleep duration, sports participation frequency, and satisfaction with sports. Participants classified with anxiety symptoms had lower scores across all HPLP-II domains and a lower total HPLP-II score than those without anxiety symptoms (97.36 ± 19.34 vs. 130.04 ± 18.36, *p* < 0.001). In the adjusted logistic regression model, higher physical activity scores were associated with lower odds of anxiety symptoms (OR = 0.756, 95% CI: 0.677–0.845, *p* < 0.001), while higher interpersonal-relationship scores were also associated with lower odds (OR = 0.877, 95% CI: 0.787–0.977, *p* = 0.017). No other variables demonstrated statistically significant adjusted associations with anxiety symptoms.

### Stress

Stress was significantly associated in the bivariate analyses with relationship status, fatigue, family socioeconomic status, smoking status, social media use, sleep duration, sports participation frequency, and satisfaction with sports. Participants classified with stress symptoms had lower scores across all HPLP-II domains and a lower total HPLP-II score than those without stress symptoms (97.49 ± 17.67 vs. 130.64 ± 19.17, *p* < 0.001). In the adjusted logistic regression model, participants reporting 3–4 h of daily social-media use had higher estimated odds of stress symptoms than those reporting ≥ 5 h/day (OR = 5.480, 95% CI: 1.054–28.482, *p* = 0.043). Satisfaction with sports was associated with lower odds of stress symptoms (OR = 0.216, 95% CI: 0.069–0.681, *p* = 0.009). Higher physical activity and stress-management scores were also associated with lower odds of stress symptoms (OR = 0.773, 95% CI: 0.677–0.882, *p* < 0.001; and OR = 0.763, 95% CI: 0.686–0.848, *p* < 0.001, respectively). Higher nutrition scores showed a small positive association with stress symptoms (OR = 1.132, 95% CI: 1.001–1.280, *p* = 0.049).

The estimates for social-media use and nutrition were relatively imprecise, as reflected by their confidence intervals, and should therefore be interpreted cautiously.

Tables 1, 2, 3, 4, 5, 6 and 7 represents the overall findings of this study.

**Table 1:** Participants’ sociodemographic characteristics and depression

Variables	Total 249 (100%)	Depression, *N* (%)		X^2^	*p* value
		Yes 106 (42.6%)	No 143 (57.4%)		
Age, year, (mean ± SD)	21.97 ± 2.40	22.15 ± 2.48	21.84 ± 2.34		
Age, category					
20 and below	71 (28.5)	27 (25.5)	44 (30.8)	2.668	0.263
21–24 years	139 (55.8)	58 (54.7)	81 (56.6)		
25 and above	39 (15.7)	21 (19.8)	18 (12.6)		
Gender					
Male	146 (58.6)	60 (56.6)	86 (60.1)	0.314	0.575
Female	103 (41.4)	46 (43.4)	57 (39.9)		
BMI, kg/m², (mean ± SD)	22.47 ± 2.84	22.43 ± 3.04	22.50 ± 2.68		
BMI, category					
Underweight	17 (6.8)	8 (7.5)	9 (6.3)	1.654	0.647
Normal weight	185 (74.3)	77 (72.6)	108 (75.5)		
Overweight	41 (16.5)	17 (16.0)	24 (16.8)		
Obese	6 (2.4)	4 (3.8)	2 (1.4)		
Relationship status					
Single	163 (65.5)	60 (56.6)	103 (72.0)	6.858	0.032^*^
In a relationship	66 (26.5)	34 (32.1)	32 (22.4)		
Married	20 (8.0)	12 (11.3)	8 (5.6)		
Living with Family					
Yes	172 (69.1)	67 (63.2)	105 (73.4)	2.976	0.085
No	77 (30.9)	39 (36.8)	38 (26.6)		
Level of fatigue					
Never	33 (13.3)	6 (5.7)	27 (18.9)	32.009	< 0.001
Low	63 (25.3)	14 (13.2)	49 (34.3)		
Moderate	123 (49.4)	66 (62.3)	57 (39.9)		
High	30 (12.0)	20 (18.9)	10 (7.0)		
Family socioeconomic status					
Low	71 (28.5)	50 (47.2)	21 (14.7)	33.998	< 0.001
Medium	154 (61.8)	52 (49.1)	102 (71.3)		
High	24 (9.6)	4 (3.8)	20 (14.0)		
Smoking status					
Yes	119 (47.8)	72 (67.9)	47 (32.9)	29.985	< 0.001
No	130 (52.2)	34 (32.1)	96 (67.1)		
Social media use, hours/day					
2 h and below	100 (40.2)	34 (32.1)	66 (46.2)	6.973	0.031^*^
3–4 h	117 (47.0)	60 (56.6)	57 (39.9)		
5 h and above	32 (12.9)	12 (11.3)	20 (14.0)		
Sleeping duration, hours/day					
6 h and below	82 (32.9)	51 (48.1)	31 (21.7)	24.329	< 0.001
7–8 h	138 (55.4)	40 (37.7)	98 (68.5)		
9 h and above	29 (11.6)	15 (14.2)	14 (9.8)		
Number of sports days/weeks					
1–2 days	16 (6.4)	4 (3.8)	12 (8.4)	38.486	< 0.001
3–4 days	55 (22.1)	21 (19.8)	34 (23.8)		
5–6 days	102 (41.0)	27 (25.5)	75 (52.4)		
7 days	76 (30.5)	54 (50.9)	22 (15.4)		
Satisfaction with sports					
Yes	179 (71.9)	57 (53.8)	122 (85.3)	29.967	< 0.001
No	70 (28.1)	49 (46.2)	21 (14.7)		

*X*^*2*^ = Chi-square test

*p* < 0.05^*^; *p* < 0.01^**^; *p* < 0.001^***^

BMI: Body Mass Index

**Table 2:** Participants’ sociodemographic characteristics and anxiety

Variables	Total 249 (100%)	Anxiety, *N* (%)		X^2^	*p* value
		Yes 106 (42.6%)	No 143 (57.4%)		
Age, year, (mean ± SD)	21.97 ± 2.40	22.11 ± 2.38	21.87 ± 2.42		
Age, category					
20 and below	71 (28.5)	25 (23.6)	46 (32.2)	2.275	0.321
21–24 years	139 (55.8)	64 (60.4)	75 (52.4)		
25 and above	39 (15.7)	17 (16.0)	22 (15.4)		
Gender					
Male	146 (58.6)	59 (55.7)	87 (60.8)	0.673	0.412
Female	103 (41.4)	47 (44.3)	56 (39.2)		
BMI, kg/m², (mean ± SD)	22.47 ± 2.84	22.95 ± 3.10	22.11 ± 2.58		
BMI, category					
Underweight	17 (6.8)	7 (6.6)	10 (7.0)	3.193	0.363
Normal weight	185 (74.3)	74 (69.8)	111 (77.6)		
Overweight	41 (16.5)	21 (19.8)	20 (14.0)		
Obese	6 (2.4)	4 (3.8)	2 (1.4)		
Relationship status					
Single	163 (65.5)	58 (54.7)	105 (73.4)	9.612	0.008^**^
In a relationship	66 (26.5)	36 (34.0)	30 (21.0)		
Married	20 (8.0)	12 (11.3)	8 (5.6)		
Living with Family					
Yes	172 (69.1)	70 (66.0)	102 (71.3)	0.798	0.372
No	77 (30.9)	36 (34.0)	41 (28.7)		
Level of fatigue					
Never	33 (13.3)	7 (6.6)	26 (18.2)	12.859	0.005^**^
Low	63 (25.3)	22 (20.8)	41 (28.7)		
Moderate	123 (49.4)	59 (55.7)	64 (44.8)		
High	30 (12.0)	18 (17.0)	12 (8.4)		
Family socioeconomic status					
Low	71 (28.5)	47 (44.3)	24 (16.8)	23.431	< 0.001
Medium	154 (61.8)	53 (50.0)	101 (70.6)		
High	24 (9.6)	6 (5.7)	18 (12.6)		
Smoking status					
Yes	119 (47.8)	68 (64.2)	51 (35.7)	19.799	< 0.001
No	130 (52.2)	38 (35.8)	92 (64.3)		
Social media use, hours/day					
2 h and below	100 (40.2)	36 (34.0)	64 (44.8)	4.449	0.108
3–4 h	117 (47.0)	58 (54.7)	59 (41.3)		
5 h and above	32 (12.9)	12 (11.3)	20 (14.0)		
Sleeping duration, hours/day					
6 h and below	82 (32.9)	41 (38.7)	41 (28.7)	10.451	0.005^**^
7–8 h	138 (55.4)	47 (44.3)	91 (63.6)		
9 h and above	29 (11.6)	18 (17.0)	11 (7.7)		
Number of sports days/weeks					
1–2 days	16 (6.4)	5 (4.7)	11 (7.7)	16.811	0.001^**^
3–4 days	55 (22.1)	20 (18.9)	35 (24.5)		
5–6 days	102 (41.0)	34 (32.1)	68 (47.6)		
7 days	76 (30.5)	47 (44.3)	29 (20.3)		
Satisfaction with sports					
Yes	179 (71.9)	62 (58.5)	117 (81.8)	16.392	< 0.001
No	70 (28.1)	44 (41.5)	26 (18.2)		

*X*^*2*^ = Chi-square test

*p* < 0.05^*^; *p* < 0.01^**^; *p* < 0.001^***^

BMI: Body Mass Index

**Table 3:** Participants’ sociodemographic characteristics and stress

Variables	Total 249 (100%)	Stress, *N* (%)		X^2^	*p* value
		Yes 109 (43.8%)	No 140 (56.2%)		
Age, year, (mean ± SD)	21.97 ± 2.40	22.14 ± 2.50	21.84 ± 2.33		
Age, category					
20 and below	71 (28.5)	27 (24.8)	44 (31.4)	2.561	0.278
21–24 years	139 (55.8)	61 (56.0)	78 (55.7)		
25 and above	39 (15.7)	21 (19.3)	18 (12.9)		
Gender					
Male	146 (58.6)	64 (58.7)	82 (58.6)	0.001	0.982
Female	103 (41.4)	45 (41.3)	58 (41.4)		
BMI, kg/m², (mean ± SD)	22.47 ± 2.84	22.66 ± 3.16	22.32 ± 2.56		
BMI, category					
Underweight	17 (6.8)	9 (8.3)	8 (5.7)	4.556	0.207
Normal weight	185 (74.3)	74 (67.9)	111 (79.3)		
Overweight	41 (16.5)	22 (20.2)	19 (13.6)		
Obese	6 (2.4)	4 (3.7)	2 (1.4)		
Relationship status					
Single	163 (65.5)	60 (55.0)	103 (73.6)	11.099	0.004^**^
In a relationship	66 (26.5)	35 (32.1)	31 (22.1)		
Married	20 (8.0)	14 (12.8)	6 (4.3)		
Living with Family					
Yes	172 (69.1)	75 (68.8)	97 (69.3)	0.007	0.935
No	77 (30.9)	34 (31.2)	43 (30.7)		
Level of fatigue					
Never	33 (13.3)	9 (8.3)	24 (17.1)	16.184	0.001^**^
Low	63 (25.3)	18 (16.5)	45 (32.1)		
Moderate	123 (49.4)	64 (58.7)	59 (42.1)		
High	30 (12.0)	18 (16.5)	12 (8.6)		
Family socioeconomic status					
Low	71 (28.5)	50 (45.9)	21 (15.0)	29.402	< 0.001
Medium	154 (61.8)	53 (48.6)	101 (72.1)		
High	24 (9.6)	6 (5.5)	18 (12.9)		
Smoking status					
Yes	119 (47.8)	71 (65.1)	48 (34.3)	23.379	< 0.001
No	130 (52.2)	38 (34.9)	92 (65.7)		
Social media use, hours/day					
2 h and below	100 (40.2)	38 (34.9)	62 (44.3)	6.718	0.035^*^
3–4 h	117 (47.0)	61 (56.0)	56 (40.0)		
5 h and above	32 (12.9)	10 (9.2)	22 (15.7)		
Sleeping duration, hours/day					
6 h and below	82 (32.9)	50 (45.9)	32 (22.9)	17.087	< 0.001
7–8 h	138 (55.4)	45 (41.3)	93 (66.4)		
9 h and above	29 (11.6)	14 (12.8)	15 (10.7)		
Number of sports days/weeks					
1–2 days	16 (6.4)	5 (4.6)	11 (7.9)	19.974	< 0.001
3–4 days	55 (22.1)	22 (20.2)	33 (23.6)		
5–6 days	102 (41.0)	33 (30.3)	69 (49.3)		
7 days	76 (30.5)	49 (45.0)	27 (19.3)		
Satisfaction with sports					
Yes	179 (71.9)	56 (51.4)	123 (87.9)	40.359	< 0.001
No	70 (28.1)	53 (48.6)	17 (12.1)		

*X*^*2*^ = Chi-square test

*p* < 0.05^*^; *p* < 0.01^**^; *p* < 0.001^***^

BMI: Body Mass Index

**Table 4:** Associations of depression, anxiety, and stress with HPLP-II scores

Variables	Spearman correlation	Yes (mean ± SD)	No (mean ± SD)	U Z	p
**Depression**					
Health responsibility	-0.451	17.74 ± 5.30	22.61 ± 4.10	3596.5 -7.100	< 0.001
Physical activity	-0.755	14.63 ± 3.62	23.97 ± 4.22	904.0 -11.895	< 0.001
Nutrition	-0.247	18.70 ± 4.80	21.29 ± 4.97	5399.5 -3.885	< 0.001
Spiritual growth	-0.692	14.24 ± 3.13	22.34 ± 4.87	1469.5 -10.890	< 0.001
Interpersonal relationships	-0.537	15.24 ± 3.46	21.13 ± 5.31	2837.0 -8.452	< 0.001
Stress management	-0.479	14.49 ± 4.60	20.43 ± 5.61	3352.5 -7.536	< 0.001
Total HPLP-II	-0.751	95.03 ± 14.75	131.77 ± 18.22	937.5 -11.820	< 0.001
**Anxiety**					
Health responsibility	−0.434	17.81 ± 5.41	22.55 ± 4.05	3741.5 -6.841	0.001^**^
Physical activity	−0.677	15.15 ± 4.70	23.58 ± 4.25	1598.0 -10.659	< 0.001
Nutrition	−0.310	18.35 ± 4.64	21.55 ± 4.93	4837.0 -4.888	< 0.001
Spiritual growth	−0.525	15.31 ± 4.32	21.55 ± 5.36	2937.0 -8.274	< 0.001
Interpersonal relationships	−0.528	15.41 ± 4.29	21.00 ± 4.98	2910.0 -8.322	< 0.001
Stress management	−0.355	15.33 ± 5.01	19.81 ± 5.92	4442.5 -5.593	< 0.001
Total HPLP-II	−0.668	97.36 ± 19.34	130.04 ± 18.36	1672.5 -10.512	< 0.001
**Stress**					
Health responsibility	-0.400	18.17 ± 5.50	22.38 ± 4.18	4084.0 -6.301	< 0.001
Physical activity	-0.654	15.46 ± 4.65	23.52 ± 4.57	1834.5 -10.294	< 0.001
Nutrition	-0.201	18.98 ± 4.87	21.13 ± 5.01	5845.5 -3.170	0.002^**^.
Spiritual growth	-0.575	15.12 ± 4.29	21.83 ± 5.13	2529.0 -9.062	< 0.001
Interpersonal relationships	-0.444	15.87 ± 4.07	20.76 ± 5.44	3698.0 -6.985	< 0.001
Stress management	-0.579	13.89 ± 3.99	21.03 ± 5.37	2501.5 -9.114	< 0.001
Total HPLP-II	-0.677	97.49 ± 17.67	130.64 ± 19.17	1620.0 -10.660	< 0.001

U, Z, p: Mann–Whitney U test

*p* < 0.05^*^; *p* < 0.01^**^; *p* < 0.001^***^

HPLP-II: Health-Promoting Lifestyle Profile II

**Table 5:** Results of logistic regression analysis, factors associated with depression

Variables	B	Wald	OR	95% CI	*p* value
Age	0.604	4.315	1.829	1.035–3.232	0.038^*^
Gender (Female, ref.)	0.835	0.298	2.306	0.115–46.179	0.585
BMI, kg/m², (mean ± SD)	-0.628	5.182	0.534	0.311–0.916	0.023^*^
Relationship status (Married, ref.)					
Single	0.791	0.103	2.206	0.018–276.050	0.748
In a relationship	1.212	0.255	3.360	0.030–371.736	0.614
Living with Family (No, ref.)					
Yes	-7.441	6.173	0.001	0.000–0.208	0.013^*^
Level of fatigue (High, ref.)					
Never	0.559	0.049	1.748	0.013–240.469	0.824
Low	-0.012	0.000	0.988	0.004–269.807	0.997
Moderate	0.151	0.006	1.163	0.024–56.529	0.939
Family socioeconomic status (High, ref.)					
Low	-1.171	1.140	0.310	0.036–2.661	0.286
Medium	-5.953	4.875	0.003	0.000–0.512	0.027^*^
Smoking status (No, ref.)					
Yes	4.903	6.087	134.689	2.740–6621.075	0.014^*^
Social media use, hours/day (5 h and above, ref.)					
2 h and below	3.148	1.222	23.297	0.088–6185.747	0.269
3–4 h	5.517	2.677	248.767	0.335–184464.197	0.102
Sleeping duration, hours/day (9 h and above, ref.)					
6 h and below	4.801	2.784	121.578	0.432–34197.600	0.095
7–8 h	0.144	0.006	1.155	0.027–49.065	0.940
Number of sports days/week (7 days, ref.)					
1–2 days	-5.844	4.196	0.003	0.000–0.777	0.041^*^
3–4 days	-6.556	5.409	0.001	0.000–0.357	0.020^*^
5–6 days	-5.674	6.046	0.003	0.000–0.316	0.014^*^
Satisfaction with sports (No, ref.)					
Yes	-1.114	0.761	0.328	0.027–4.012	0.383
Health responsibility	0.033	0.072	1.033	0.813–1.313	0.788
Physical activity	-0.773	9.954	0.462	0.286–0.746	0.002^**^
Nutrition	0.153	1.491	1.166	0.911–1.490	0.222
Spiritual growth	-0.427	2.351	0.653	0.378–1.126	0.125
Interpersonal relationships	-0.372	3.736	0.690	0.473–1.005	0.053
Stress management	-0.324	4.303	0.723	0.532–0.982	0.038^*^

Binary Logistic Regression

^*^p < 0.05, ^**^p < 0.01, ^***^p < 0.001; OR, odds ratio; CI, confident interval

BMI: Body Mass Index

**Table 6:** Results of logistic regression analysis, factors associated with anxiety

Variables	B	Wald	OR	95% CI	*p* value
Age	-0.010	0.013	0.990	0.826–1.185	0.911
Gender (Female, ref.)	-0.522	1.293	0.594	0.242–1.459	0.256
BMI, kg/m², (mean ± SD)	0.122	2.220	1.130	0.962–1.328	0.136
Relationship status (Married, ref.)					
Single	0.249	0.092	1.283	0.258–6.389	0.761
In a relationship	0.859	0.978	2.360	0.430–12.942	0.323
Living with Family (No, ref.)					
Yes	-0.541	1.294	0.582	0.229–1.479	0.255
Level of fatigue (High, ref.)					
Never	0.314	0.091	1.368	0.178–10.527	0.763
Low	1.415	2.338	4.117	0.671–25.253	0.126
Moderate	0.324	0.186	1.382	0.317–6.030	0.667
Family socioeconomic status (High, ref.)					
Low	-0.526	1.199	0.591	0.231–1.515	0.274
Medium	-0.640	0.587	0.527	0.103–2.709	0.444
Smoking status (No, ref.)					
Yes	0.091	0.043	1.095	0.462–2.594	0.837
Social media use, hours/day (5 h and above, ref.)					
2 h and below	0.379	0.262	1.461	0.343–6.225	0.609
3–4 h	0.687	0.930	1.988	0.492–8.029	0.335
Sleeping duration, hours/day (9 h and above, ref.)					
6 h and below	-0.465	0.399	0.628	0.149–2.657	0.528
7–8 h	-0.745	1.127	0.475	0.120–1.878	0.288
Number of sports days/week (7 days, ref.)					
1–2 days	-0.124	0.019	0.883	0.148–5.261	0.892
3–4 days	-0.994	2.779	0.370	0.115–1.191	0.095
5–6 days	-0.414	0.597	0.661	0.232–1.888	0.440
Satisfaction with sports (No, ref.)					
Yes	-0.159	0.100	0.853	0.317–2.294	0.752
Health responsibility	-0.073	2.248	0.930	0.845–1.023	0.134
Physical activity	-0.280	24.527	0.756	0.677–0.845	< 0.001
Nutrition	-0.025	0.257	0.975	0.884–1.076	0.612
Spiritual growth	-0.024	0.168	0.977	0.872–1.093	0.682
Interpersonal relationships	-0.131	5.701	0.877	0.787–0.977	0.017^*^
Stress management	0.039	0.846	1.040	0.957–1.130	0.358

Binary Logistic Regression

^*^p < 0.05, ^**^p < 0.01, ^***^p < 0.001; OR, odds ratio; CI, confident interval

BMI: Body Mass Index

**Table 7:** Results of logistic regression analysis, factors associated with stress

Variables	B	Wald	OR	95% CI	*p* value
Age	0.149	2.120	1.161	0.950–1.419	0.145
Gender (Female, ref.)	1.026	3.103	2.791	0.891–8.744	0.078
BMI, kg/m², (mean ± SD)	-0.171	3.111	0.843	0.697–1.019	0.078
Relationship status (Married, ref.)					
Single	-1.504	2.470	0.222	0.034–1.450	0.116
In a relationship	-0.874	0.804	0.417	0.062–2.821	0.370
Living with Family (No, ref.)					
Yes	-0.185	0.120	0.831	0.293–2.361	0.729
Level of fatigue (High, ref.)					
Never	0.109	0.011	1.115	0.145–8.563	0.917
Low	-0.516	0.310	0.597	0.097–3.671	0.578
Moderate	0.144	0.035	1.154	0.257–5.182	0.851
Family socioeconomic status (High, ref.)					
Low	-0.877	2.730	0.416	0.147–1.177	0.098
Medium	-0.824	0.811	0.439	0.073–2.638	0.368
Smoking status (No, ref.)					
Yes	1.028	3.638	2.795	0.972–8.035	0.056
Social media use, hours/day (5 h and above, ref.)					
2 h and below	1.657	3.516	5.243	0.928–29.626	0.061
3–4 h	1.701	4.092	5.480	1.054–28.482	0.043^*^
Sleeping duration, hours/day (9 h and above, ref.)					
6 h and below	1.385	2.642	3.996	0.752–21.243	0.104
7–8 h	-0.061	0.006	0.941	0.196–4.516	0.940
Number of sports days/week (7 days, ref.)					
1–2 days	-0.246	0.055	0.782	0.100–6.133	0.815
3–4 days	-0.369	0.323	0.691	0.193–2.471	0.570
5–6 days	-0.255	0.178	0.775	0.238–2.530	0.673
Satisfaction with sports (No, ref.)					
Yes	-1.532	6.839	0.216	0.069–0.681	0.009^**^
Health responsibility	-0.035	0.353	0.966	0.861–1.083	0.552
Physical activity	-0.258	14.509	0.773	0.677–0.882	< 0.001
Nutrition	0.124	3.876	1.132	1.001–1.280	0.049^*^
Spiritual growth	-0.018	0.058	0.982	0.849–1.137	0.810
Interpersonal relationships	-0.003	0.003	0.997	0.891–1.114	0.953
Stress management	-0.271	25.004	0.763	0.686–0.848	< 0.001

Binary Logistic Regression

^*^p < 0.05, ^**^p < 0.01, ^***^p < 0.001; OR, odds ratio; CI, confident interval

BMI: Body Mass Index

## Discussion

The present study examined associations between lifestyle factors and PD among university athletes in Bangladesh. Depression (42.6%), anxiety (42.6%), and stress (43.8%) were prevalent, indicating a substantial mental health burden in this population and extending the limited evidence from South Asian university athlete settings. Recent international evidence similarly indicates that PD symptoms are common among university athletes, although estimates vary considerably according to population, sex, sport, competitive context, measurement instrument, and symptom thresholds [8, 9]. Findings of this study suggest an interrelationship between lifestyle behaviors and psychological well-being; however, the observed associations should be interpreted as correlational rather than causal given the cross-sectional design.

The high prevalence observed in this study should be interpreted in the context of the multiple and potentially competing demands experienced by university athletes. Beyond academic responsibilities, university athletes may face training and competition demands, performance expectations, injury concerns, time constraints, and uncertainty about their academic and sporting careers. These pressures may interact with social circumstances and health behaviors to shape psychological well-being. Nevertheless, the cross-sectional design does not establish whether these circumstances preceded psychological symptoms or were themselves influenced by distress. Accordingly, the observed prevalence should be interpreted as an estimate of symptom burden rather than evidence that participation in university sport increases psychological morbidity [9, 20].

A prominent finding was the consistently poorer health-promoting lifestyle profile among participants reporting depression, anxiety, or stress symptoms. In the unadjusted comparisons, participants with psychological symptoms had lower total HPLP-II scores and lower scores across all six lifestyle domains, suggesting that psychological distress coexisted with a broader pattern of less favorable health-promoting behaviors. This pattern is broadly consistent with evidence indicating that PD among young adults may cluster with reduced engagement in health-promoting behaviors and psychosocial resources [7, 10]. The relationship is likely complex and potentially bidirectional: psychological symptoms may adversely affect motivation, self-regulation, energy, and engagement in daily health behaviors, while unfavorable behavioral and psychosocial patterns may coexist with or contribute to greater psychological burden. Importantly, however, several domain-specific associations were attenuated after multivariable adjustment, indicating that the observed overall lifestyle differences should not be interpreted as evidence that every lifestyle dimension was independently associated with psychological symptoms. The cross-sectional design further precludes establishing temporal direction.

Among the individual lifestyle domains, physical activity demonstrated the most consistent independent association, with higher scores associated with lower odds of depression, anxiety, and stress. This pattern is consistent with the broader literature reporting associations between greater physical activity and better mental health among young adults and university athletes [21]. Possible explanations include improved emotional well-being, greater perceived competence and self-efficacy, and opportunities for social engagement associated with an active lifestyle. Nevertheless, these findings represent observational associations rather than evidence of a protective effect. Reverse causality remains plausible, as participants experiencing fewer psychological symptoms may have greater motivation, energy, and capacity to engage in regular physical activity.

The adjusted analyses further indicated that particular lifestyle dimensions were associated with specific psychological outcomes. Higher stress-management scores were associated with lower odds of depression and stress, whereas higher interpersonal-relationship scores were associated with lower odds of anxiety. These findings are broadly consistent with literature highlighting the importance of adaptive coping and positive interpersonal functioning in psychological well-being [7, 22, 23]. Individuals who engage more frequently in effective stress-management behaviors may be better able to manage academic, sporting, and everyday demands, while positive interpersonal functioning may be associated with greater emotional and social resources. In contrast, health responsibility and spiritual growth were not independently associated with depression, anxiety, or stress after adjustment, suggesting that lower scores across these domains in the unadjusted comparisons did not translate into independent associations. The small positive association between nutrition and stress was statistically borderline and should be interpreted cautiously, particularly because the direction of the association is not readily explained as a harmful effect of healthier nutritional behavior. Residual confounding, reverse causality, or behavioral changes accompanying psychological stress may account for this finding.

Greater satisfaction with sports was also associated with lower odds of stress, suggesting that the subjective experience of sport participation may be relevant to psychological well-being in addition to participation frequency. This finding is broadly consistent with research indicating that positive sporting experiences may be associated with better psychological outcomes, whereas negative experiences within sporting contexts may contribute to psychological strain [24]. However, the direction of this association cannot be determined from the present data: participants experiencing lower stress may perceive their sporting experience more positively, rather than greater sports satisfaction necessarily leading to reduced stress. Accordingly, sports satisfaction should be interpreted as a potentially important correlate of psychological well-being rather than an established protective factor.

Several sociodemographic and behavioral characteristics were independently associated with psychological symptoms, although these findings should be interpreted as associations rather than causal effects. The association between older age and depressive symptoms may reflect the cumulative academic, athletic, and psychosocial demands students experience as they progress through university, including greater concerns about academic performance, career transitions, and sporting expectations [6]. Higher BMI was associated with lower odds of depressive symptoms; however, this finding requires cautious interpretation, particularly in university athletes, among whom BMI may reflect differences in body composition and physical characteristics rather than adiposity alone. Living with family and socioeconomic circumstances may influence psychological well-being through differences in social support, financial security, and access to resources that buffer psychological distress, potentially explaining their inverse associations with depressive symptoms. The substantially elevated odds of depression among smokers are consistent with the observed association between tobacco use and poorer mental health; however, the wide confidence interval indicates considerable imprecision in the estimate and warrants cautious interpretation [2]. Likewise, the apparently strong associations observed for some categories of sports participation should be interpreted cautiously because of their imprecise estimates. For stress, the association with social-media use may reflect the complex relationship between digital engagement, social comparison, information overload, and psychological stress, whereas greater satisfaction with sports may indicate that positive enjoyment and perceived fulfillment from sporting participation are associated with a more favorable psychosocial experience [7, 16]. Overall, these findings suggest that the social and behavioral context surrounding university athletes may contribute to psychological well-being, although longitudinal studies with larger and more diverse samples are needed to clarify the direction and underlying mechanisms of these associations.

Importantly, these findings should not be interpreted as demonstrating that particular lifestyle characteristics protect university athletes against depression, anxiety, or stress. Because the outcomes were relatively common in this sample, the odds ratios may differ materially from corresponding prevalence ratios and should not be interpreted as risk ratios. The DASS-21 is a validated self-report instrument for assessing the severity of depressive, anxiety, and stress symptoms and does not establish clinical psychiatric diagnoses. Accordingly, differences in assessment instruments, outcome definitions and thresholds, sampling strategies, and population characteristics may contribute to variation in reported prevalence estimates across studies and should be considered when comparing national and international settings.

Overall, the findings support a multidimensional understanding of psychological health among university athletes in Bangladesh. Rather than identifying a single explanatory or modifiable determinant, the results suggest that psychological symptoms coexist with differences in physical activity, stress-management behaviors, interpersonal functioning, satisfaction with sports participation, and selected sociodemographic and behavioral characteristics. This pattern is consistent with contemporary conceptualizations of university athlete mental health, in which psychological well-being is shaped by interacting individual, behavioral, social, sporting, and broader contextual factors rather than a single pathway. However, the cross-sectional nature of the study prevents determination of the temporal ordering or relative contribution of these factors, underscoring the need for longitudinal research to clarify how these characteristics interact over time.

### Strengths and limitations

The study has several strengths, including its focus on an understudied university athlete population in Bangladesh, assessment of multiple psychological outcomes alongside a broad range of lifestyle domains, and simultaneous consideration of sociodemographic, behavioral, and sport-related characteristics. The use of prespecified logistic regression models, reporting of 95% confidence intervals, and reporting of model diagnostics details strengthen the analytical transparency.

Several limitations warrant consideration. First, the cross-sectional design prevents establishing temporality or causal direction and leaves reverse causality plausible, particularly for physical activity, stress management, smoking, social-media use, and sports activities. Second, convenience sampling may have introduced selection bias and limits generalizability beyond the participating universities and athletes. Third, reliance on self-reported measures may introduce recall, reporting, and social-desirability bias, while dichotomizing DASS-21 scores may reduce statistical power and information. Fourth, using both online and offline data collection may have introduced differences in privacy, digital access, response behavior, or willingness to disclose psychological symptoms, although the study was not designed to formally evaluate recruitment-mode effects. Fifth, residual confounding is possible because important variables including previous PD history, injury status, competitive level, alcohol use, sleep quality, and academic workload were not comprehensively assessed. These factors may influence both psychological symptoms and lifestyle behaviors and could therefore attenuate or exaggerate observed associations. Finally, several regression estimates were highly imprecise, suggesting potential sensitivity to model diagnostics. These estimates should consequently be considered hypothesis-generating rather than definitive.

### Implications

The findings have several practical implications. Universities and sports programs should consider psychological well-being as part of routine athlete support rather than treating mental health as separate from athletic and academic development. Screening and early identification may be particularly valuable where substantial symptom burden is present, with referral pathways to appropriately qualified mental-health professionals. Health-promotion initiatives could incorporate physical activity, stress-management skills, interpersonal support, and positive sporting environments as components of a broader, integrated approach; however, these components should be evaluated prospectively rather than assumed to produce therapeutic effects based on the present findings. Future research should prioritize multicenter prospective studies with representative sampling, repeated assessment of psychological symptoms and lifestyle behaviors, and measurement of clinically relevant confounders such as prior mental health history, injury, training load, competitive level, sleep quality, substance use, and academic workload. Intervention studies are subsequently needed to determine whether modifying these potentially relevant factors produces clinically meaningful improvements in university athlete mental health.

## Conclusion

Depressive, anxiety, and stress symptoms were common among university athletes in Bangladesh, indicating a substantial burden of psychological symptoms in this population. Psychological symptoms were associated with a broad range of lifestyle, sociodemographic, behavioral, and sport-related characteristics. Among lifestyle domains, physical activity showed the most consistent association across depression, anxiety, and stress, while stress management, interpersonal relationships, and sport-related characteristics were associated with selected psychological outcomes. Collectively, these findings support a multidimensional understanding of psychological well-being among university athletes, in which psychological symptoms coexist with differences in behavioral, interpersonal, sporting, and contextual characteristics. However, given the cross-sectional design, these findings should be interpreted as correlational rather than causal and should not be taken to indicate that any identified characteristic is a determinant or protective factor.

## Supplementary Information

Below is the link to the electronic supplementary material.

Supplementary Material 1

Supplementary Material 2

## Data Availability

The data sets used and analyzed in this study are available from the corresponding author on reasonable request.

## Declarations

### Participants and public involvement

Participants and/or the public were not involved in the design, conduct, reporting, or dissemination plans of this research.

## Abbreviations

AEBQ: Adult Eating Behavior Questionnaire
BMI: Body Mass Index
CI: Confidence Interval
DALYs: Disability–Adjusted Life Years
DASS: 21–Depression, Anxiety, and Stress Scale–21
HPLP: II–Health–Promoting Lifestyle Profile II
ICC: Intraclass Correlation Coefficient
IPAQ: International Physical Activity Questionnaire
JUST: Jashore University of Science and Technology
OR: Odds Ratio
PD: Psychological Distress
SPSS: Statistical Package for the Social Sciences
