# Supplementary material for: Prevalence and associated factors of depression, anxiety, and stress among university athletes in Bangladesh
Source: Discov Ment Health. 2026 Nov 6;6(1):226. doi: 10.1007/s44192-026-00660-9 (PMC13768422; doi:10.1007/s44192-026-00660-9)
Supplement: Supplementary file 2 [file 44192_2026_660_MOESM2_ESM.docx]

**Supplementary Table 1. Model diagnostics and discrimination measures**

| **Diagnostic measure** | **Depression** | **Anxiety** | **Stress** |
| --- | --- | --- | --- |
| Total analytical sample, n | 249 | 249 | 249 |
| Events, n (%) | 106 (42.6) | 106 (42.6) | 109 (43.8) |
| Non-events, n (%) | 143 (57.4) | 143 (57.4) | 140 (56.2) |
| Estimated model parameters, n | 26 | 26 | 26 |
| Events per estimated parameter | 4.08 | 4.08 | 4.19 |
| VIF range | 1.024–2.387 | 1.024–2.387 | 1.024–2.387 |
| Minimum tolerance | 0.419 | 0.419 | 0.419 |
| Hosmer–Lemeshow χ² | 1.459 | 10.322 | 9.621 |
| df | 8 | 8 | 8 |
| *p* value | 0.993 | 0.217 | 0.293 |
| AUC | 0.995 | 0.870 | 0.926 |
| 95% CI for AUC | 0.990–1.000 | 0.821–0.919 | 0.889–0.964 |
| Marginal sparse cells | None identified | None identified | None identified |
| Model convergence warning | None | None | None |

*Footnote:* VIF, variance inflation factor; AUC, area under the receiver operating characteristic curve; CI, confidence interval. For the Hosmer–Lemeshow test, p > 0.05 indicates no evidence of lack of model fit. No convergence warnings were observed.
